# Novel curriculum of smoking cessation for dental students

**DOI:** 10.1186/1617-9625-12-S1-A2

**Published:** 2014-06-06

**Authors:** Takashi Hanioka

**Affiliations:** 1 Dept. of Preventive and Public Health Dentistry, Fukuoka Dental College, Fukuoka, 814-0193, Japan

## Background

Dental professionals have not fully embraced opportunities for tobacco intervention. The aim of my presentation is to suggest new strategy of curriculum to enhance smoking cessation intervention based on the novel findings that may useful under the universal health insurance system in Japan.

## Materials and methods

Reviewed literatures regarding tobacco interventions by dental professionals and recent studies regarding oral microbiology, and surveyed dentists and dental patients to clarify the possibility of our strategy.

## Results

The literature review regarding progression of the global dental tobacco interventions identified significant barriers such as lack of reimbursement and training for implementation to dental practice and dissemination and undergraduate education. Recent findings regarding the effects of smoking cessation on oral biofilms and those of tobacco extracts on virulence factor of periodontal pathogens would break the ice to enforce dental tobacco intervention in Japan. The studies that was conducted in dental clinics revealed that tobacco intervention for prevention of progression of dental disease and improvement of the effects of dental treatments that have potential coverage of the universal health insurance system in Japan were strongly supported by dentists while intervention services for prevention of oral diseases was strongly supported by dental patients.

## Conclusions

Education for dental students regarding tobacco intervention based on the effects on dental treatments would be promising strategy that reinforces those for oral and overall health in Japan.

